# Supplementary material for: Dietary amino acids promote glucagon-like hormone release to generate global calcium waves in adipose tissues in Drosophila
Source: Nat Commun. 2025 Jan 2;16:247. doi: 10.1038/s41467-024-55371-y (PMC11696257; doi:10.1038/s41467-024-55371-y)
Supplement: Supplementary file 1 — Supplementary Information [file 41467_2024_55371_MOESM1_ESM.pdf]

Supplementary Information

Dietary Amino Acids Promote Glucagon-like Hormone Release to Generate Global Calcium Waves in Adipose Tissues

Muhammad Ahmad[1†], Shang Wu[2†], Shengyao Luo[3], Wenjia Shi[4], Xuan Guo[5], Yuansheng Cao[6,*], Norbert Perrimon[1,7]* , Li He[2,*]

1. Department of Genetics, Harvard Medical School, Boston, Massachusetts, 02115, USA
2. The First Affiliated Hospital of USTC, Division of Life Sciences and Medicine, University of Science and Technology of China, Hefei, Anhui 230000, China
3. Yuanpei College, Peking University, 100871 Beijing, China.
4. Department of Applied Physics, Xi'an University of Technology, Xi'an 710048, Shaanxi, China.
5. Life Science Institute, Jinzhou Medical University, Jinzhou, Liaoning 121001, China
6. Department of Physics, Tsinghua University, 100084, Beijing, China
7. Howard Hughes Medical Institute, Harvard Medical School, Boston, Massachusetts, 02115, USA

[†]These authors contributed equally to this work

*For Correspondence:

lihe19@ustc.edu.cn

perrimon@genetics.med.harvard.edu

yscao@tsinghua.edu.cn

Supplementary Methods

Computational Model and Numerical Method

Our mathematical method employs a simplified version of a receptor-operator calcium channel model to describe Ca^{2+} dynamics within the fat body[1]. We consider the Ca^{2+} concentration in two key spatiotemporal variables: the cytoplasm (denoted as C) and in the endoplasmic reticulum (ER, denoted as C_e). These variables represent the core aspect of calcium oscillation and wave propagation and are assumed to be homogeneous within a single cell. A set of ordinary differential equations (ODEs) governs their behavior[1]. Spatial coupling between neighboring cells is captured by Ca^{2+} diffusion. For simplicity, we do not explicitly track individual cells. Instead, we assume C and C_e are spatially continuous throughout the simulation domain. Consequently, C and C_e are described by a set of partial differential equations (PDEs).

For the intracellular Ca^{2+} signaling, AKH binding to AkhR triggers the activation of phospholipase C (PLC) and the subsequent generation of IP3. IP3 diffuses throughout the cytoplasm and activates the IP3 receptor (IP3R) on the ER membrane, inducing Ca^{2+} release from the ER to the cytoplasm. The Ca^{2+} release influx can also further be stimulated by Ca^{2+} already in the cytoplasm, forming a positive feedback mechanism that leads to the surge in Ca^{2+} concentration. The Ca^{2+} release from ER to the cytoplasm is given by:

$$J_{IPR} = k_f P_o (C_e - C),$$

where k_f refers to the maximal rate of Ca^{2+} release. P_o denotes the open probability of IP3R, which is determined by C, the IP_3 concentration p and the fraction of inactivated receptors y, explicitly written as[2]:

$$P_o = \left(\frac{p(1-y)C}{(p+K_1)(C+K_2)} \right)^3,$$

where K_1 is the rate constant characterizing IP3 binding to IP3R and K_2 is the rate constant characterizing Ca^{2+} binding.

Following Ca^{2+} excitation, countering mechanisms restore cytoplasmic Ca^{2+} concentration to its resting level. SERCA pumps on the ER membrane play a negative feedback role by actively transporting Ca^{2+} from the cytoplasm back into the ER. This flux (J_{SERCA}) can be described using a Hill function:

$$J_{SERCA} = \frac{V_s C^2}{K_s^2 + C^2},$$

where V_s is the maximum rate of the SERCA pump and K_s is the half-activation constant. In addition, we incorporate three Ca^{2+} fluxes: influx to the cell (J_{in}), efflux (J_{pm}), and leak from the ER into the cytoplasm (J_{leak}). The additional terms are necessary for maintaining a steady state when p = 0, i.e., when the IP3R are all inactive. J_{leak} represents the unspecific leakage of Ca^{2+} from the ER to the cytoplasm and is assumed to be proportional to the concentration difference between two compartments: $J_{leak} = k_{leak}(C_e - C)$. J_{pm} describes the active removal of Ca^{2+} from the cytoplasm by pumps on the plasma membrane (e.g., PMCA). This efflux is modeled using a Hill function: $J_{pm} = \frac{V_p^2 C^2}{K_p^2 + C^2}$. Consistent with previous studies[2, 3], the influx from extracellular spaces J_{in} is assumed to be a simple linear function of the IP3 concentration p: $J_{in} = \alpha_0 + \alpha_1 p$. In the above fluxes, $V_p, K_p, k_{leak}, \alpha_0, \alpha_1$ are dynamics constants.

Put the relevant fluxes together, the PDEs for C and C_e are:

$$\frac{dC}{dt} = J_{IPR} + J_{leak} - J_{serca} + J_{in} - J_{pm} + D_c \nabla^2 C, \quad (1)$$

$$\frac{dC_e}{dt} = -\gamma(J_{IRP} + J_{leak} - J_{serca}), \quad (2)$$

where γ accounts for the volume difference between cytoplasm and ER. The diffusion of Ca^{2+} in the cytoplasm through gap junctions is described by $D_c \nabla^2 C$, where D_c denotes the effective diffusion coefficient of Ca^{2+}. $D_c = 0$ when the gap junctions are knocked down

The concentration of IP3 (p) is dynamically regulated by a complex network of metabolic reactions modulated by the extracellular signaling molecules, e.g., AKH for our model. Due to the rapid nature of receptor signaling events, we assume p reaches a fast equilibrium. Therefore, p is modeled as linearly proportional to the extracellular AKH concentration C_A:

$$p = k_A C_A + p_0.$$

Here p_0 is the basic IP3 concentration when there is no stimulation of AKH, and k_A is the AKH activation coefficient. In the larvae, the dynamics of C_A is described by the diffusion-reaction-advection equation:

$$\frac{dC_A}{dt} = -\mu C_A - v_{tr}\, \partial C_A / \partial x + D_a \nabla^2 C_A. \quad (3)$$

Here, μ is the AKH degradation rate. v_{tr} represents the effective advection speed of AKH through lymphatic circulation and D_a denotes the diffusion coefficient. The above equation is accompanied by a source term of AKH secretion at APCs as a boundary condition.

Besides the deterministic part, we add a random noise $\sigma\eta(x, y, t)$ directly to the differential equations of C and C_A, where σ represents the fluctuation intensity constants. σ could be spatially uniform or non-uniform. The term $\eta(x, y, t)$ represent a standard white noise, which satisfies:

$$\langle \eta(x, y, t) \rangle = 0,$$

$$\langle \eta(x_1, y_1, t_1)\eta(x_1, y_2, t_2) \rangle = 2\delta(t_1 - t_2)\delta(x_1 - x_2)\delta(y_1 - y_2),$$

where $\langle \cdot \rangle$ means the ensemble average, and $\delta(s) = 1$ only when $s = 0$, otherwise $\delta(s) = 0$.

Equations (1-3) constitute our main model. They are solved numerically using the Euler finite-difference method in a two-dimensional plane, while the Crank–Nicolson scheme is used to calculate the advection term convergently. No outflow boundary conditions are implemented. Model parameters are listed in

Supplementary Table 3, and all MATLAB codes in the simulations are fully disclosed.

References

1. Dupont, G., Falcke, M., Kirk, V., Sneyd, J. *Models of Calcium Signalling*, Vol. 43. (SpringerLink, 2016).
2. Kummer, U. *et al.* Switching from simple to complex oscillations in calcium signaling. *Biophys J* **79**, 1188-1195 (2000).
3. Dupont, G. & Goldbeter, A. One-pool model for Ca2+ oscillations involving Ca2+ and inositol 1,4,5-trisphosphate as co-agonists for Ca2+ release. *Cell Calcium* **14**, 311-322 (1993).

Supplementary Figures

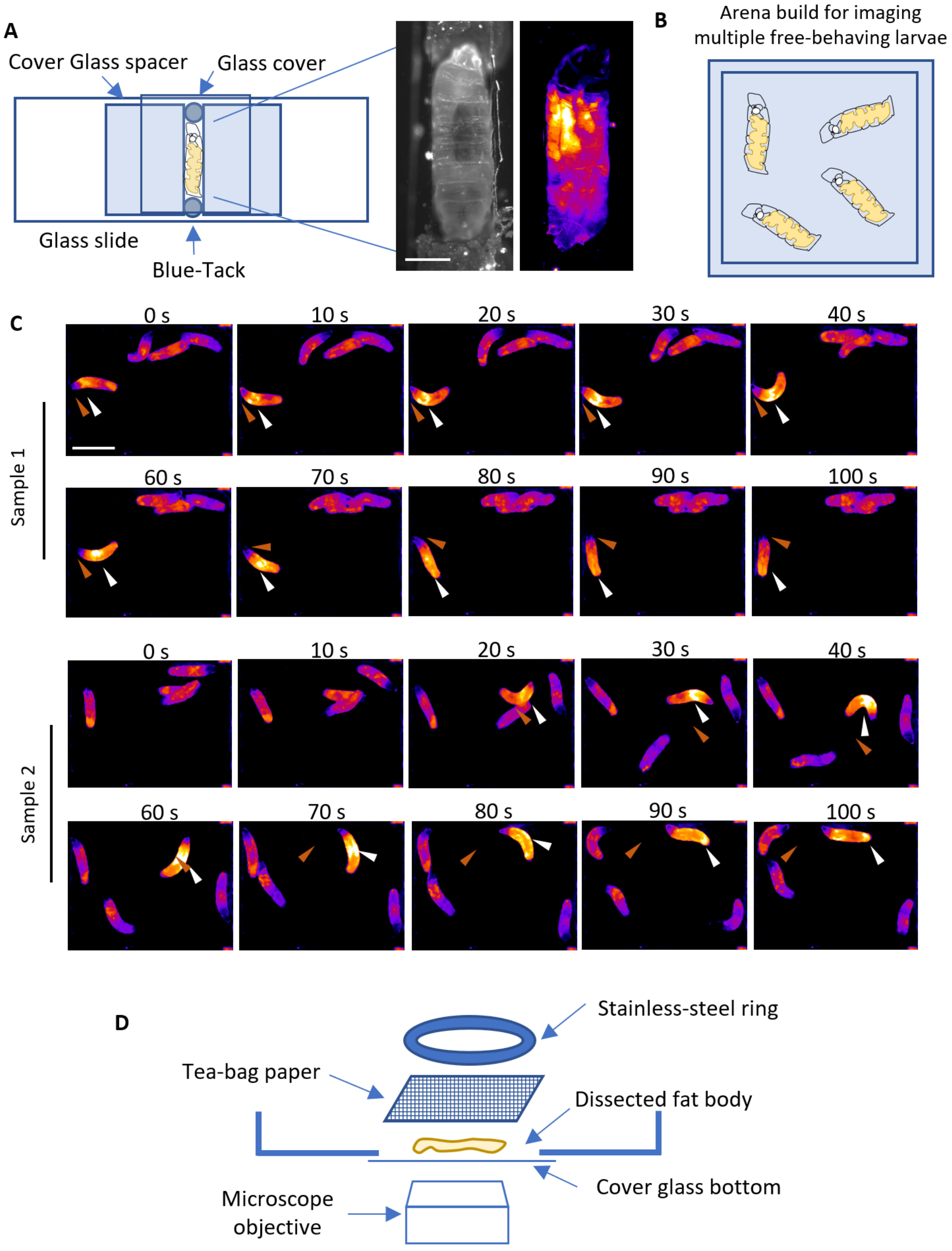

Supplementary Figure 1. Setups for imaging the *in vivo* and *in vitro* ICWs in the fat body of fly larvae. (A) 3rd instar larvae were placed in a narrow glass channel to restrict mobility. Adipose tissue-specific Ca^{2+} activities were visualized in *Fb>UAS-GCaMP5G* animals. Two common fat body-specific Gal4 drivers, *Fb-*

Gal4 and *Lpp-Gal4*, are employed for fat-specific expression in this study. No difference in Ca^{2+} dynamics was observed when using the two Gal4 drivers to express GCaMP5G in the fat body. **(B)** An arena fitting the imaging field of a fluorescent dissection microscope was generated by 3D printing. A glass was put on the top of the arena to prevent the escape of larvae. **(C)** Ca^{2+} activity in the fat body of free-behaving 3rd instar larvae was revealed in *Fb-Gal4>GCaMP5G*. The head of the larva was indicated by the orange arrow and the maximum of ICW was indicated by the white arrow. **(D)** A schematic illustration of the setup for *ex vivo* imaging of dissected fat body. Scale bars, 1 mm (**A**), 5 mm (**C**).

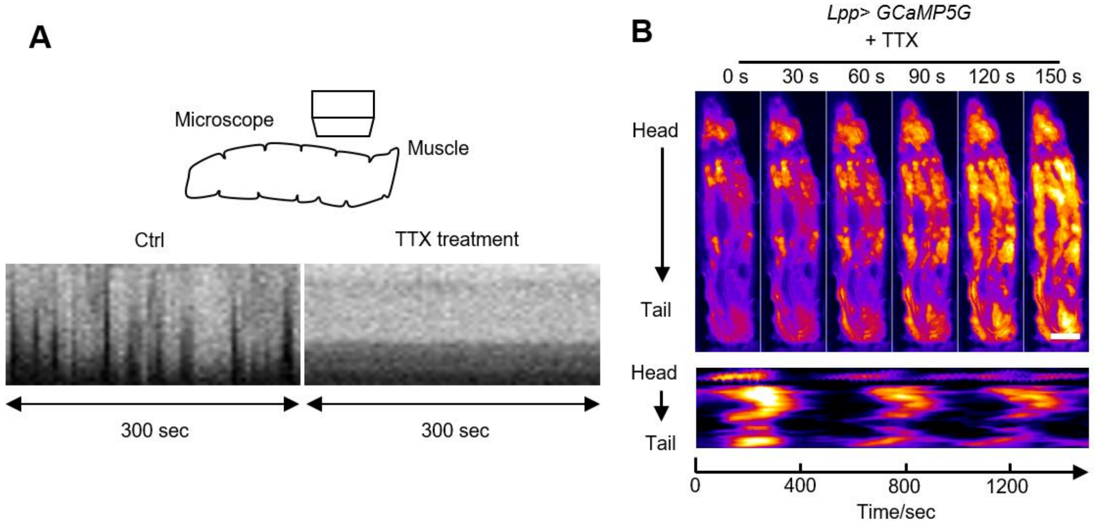

Supplementary Figure 2. Global ICWs are not caused by muscle contractions. (A) 3rd instar larvae were fed with lipid food containing 5% sucrose and 300 μM TTX for 30 mins to paralyze their skeleton muscle. A Kymograph of the cuticle through light-field imaging showed that the muscle contraction is completely stopped. Notably, we found that TTX does not affect heart contraction which is consistent with the previous report. The result was repeated in 3 independent experiments. (**B**) The ICWs in the 3rd instar larvae were imaged with TTX feeding. The data showed that inhibition of muscle contraction by feeding TTX does not affect the global ICWs *in vivo*. The result was repeated in 3 independent experiments. Scale bars, 500 μm (**B**).

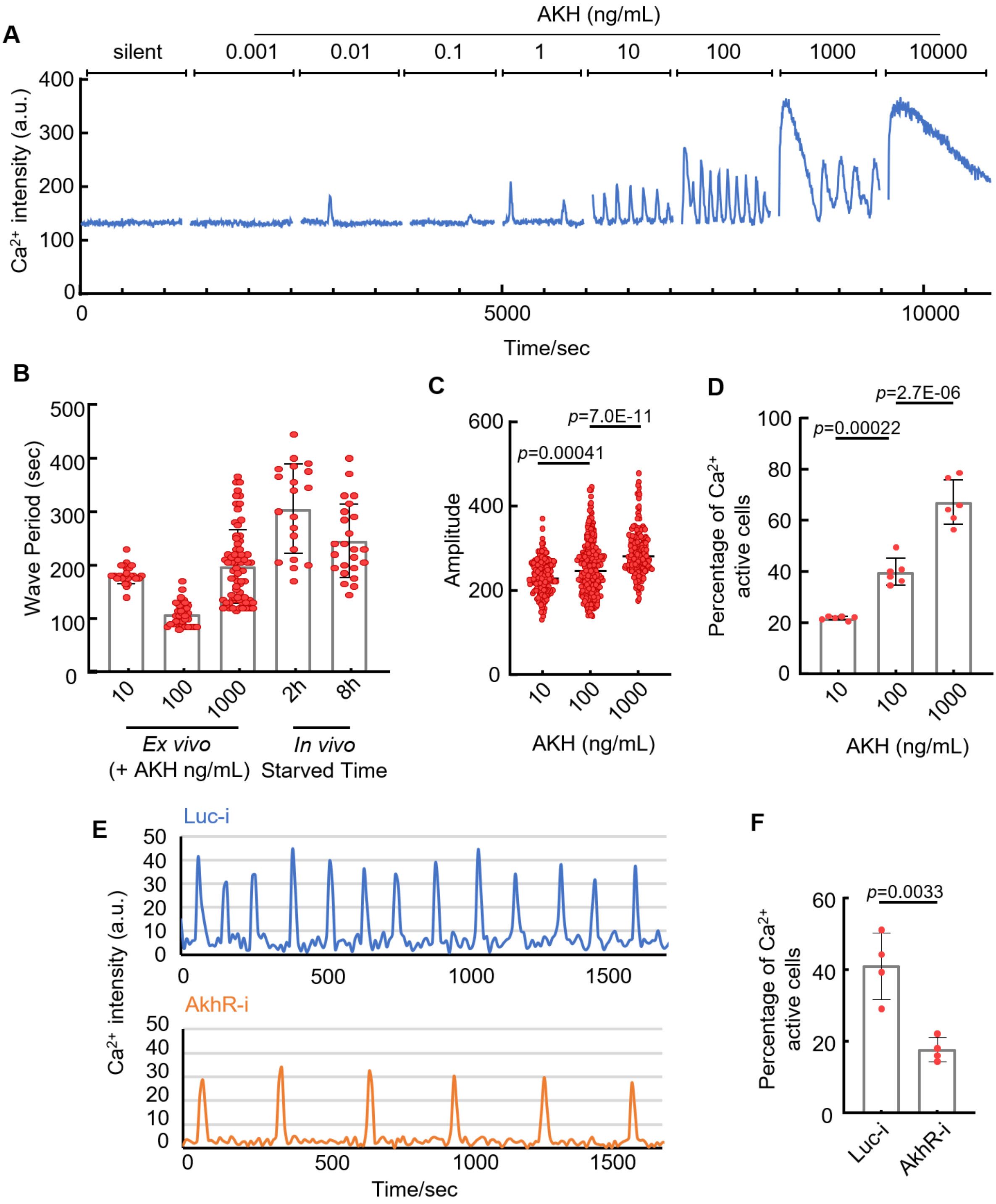

Supplementary Figure 3. Ca^{2+} oscillation in the fat cells depends on the concentration of applied AKH and the expression level of AkhR. (A) 3rd instar larvae expressing *GCaMP5G-T2A-mRuby* were treated with different concentrations of synthesized AKH. A representative trace of Ca^{2+} dynamics from a small region containing 1-2 fat cells was plotted. (**B**) The *ex vivo* and *in vivo* oscillation periods of ICWs in the 3rd instar larval fat body. Early 3rd instar digging larvae with the fat body-specific expression of GCaMP5G-T2A-mRuby were collected from normal food, washed, and transferred onto a 2% agarose (in water) plate for starvation.

After 2 h or 8 h of starvation, more than 10 larvae were kept on 2% agarose and imaged for 20 minutes under free-behavior conditions. The period of the ICWs from head to tail of the larvae was calculated in ImageJ. Data was pooled from 3 independent experiments. N = 24, 34, 74 oscillations (Ex vivo), 20, 24 oscillations (In vivo). **(C-D)** The amplitude and percentage of cells with ICWs in cultured fat bodies treated with different concentrations of AKH were quantified. Data was pooled from 3 independent experiments. *N* = 165, 271, 187 oscillations (**C**). *N* = 6, 6, 6 fat bodies (**D**). **(E-F)** Fat body expressing *GCaMP5G-T2A-mRuby* together with Ctrl (*luciferase-RNAi*) or *AkhR-RNAi* were treated with 100 ng mL^{-1} synthesized AKH peptide. Representative traces of Ca^{2+} dynamics from a single fat cell was plotted. *AkhR-RNAi* significantly reduced the Ca^{2+} oscillation frequency and the percentage of cells with the Ca^{2+} oscillation. *N* = 4, 4 fat bodies (**F**). Data were plotted as mean ± SD. Statistical significance was determined using Ordinary one-way ANOVA with Dunnett's multiple comparisons test in (**C**, **D**), the unpaired Student's t-test with two-tailed was used in (**F**). Source data are provided as a Source Data file.

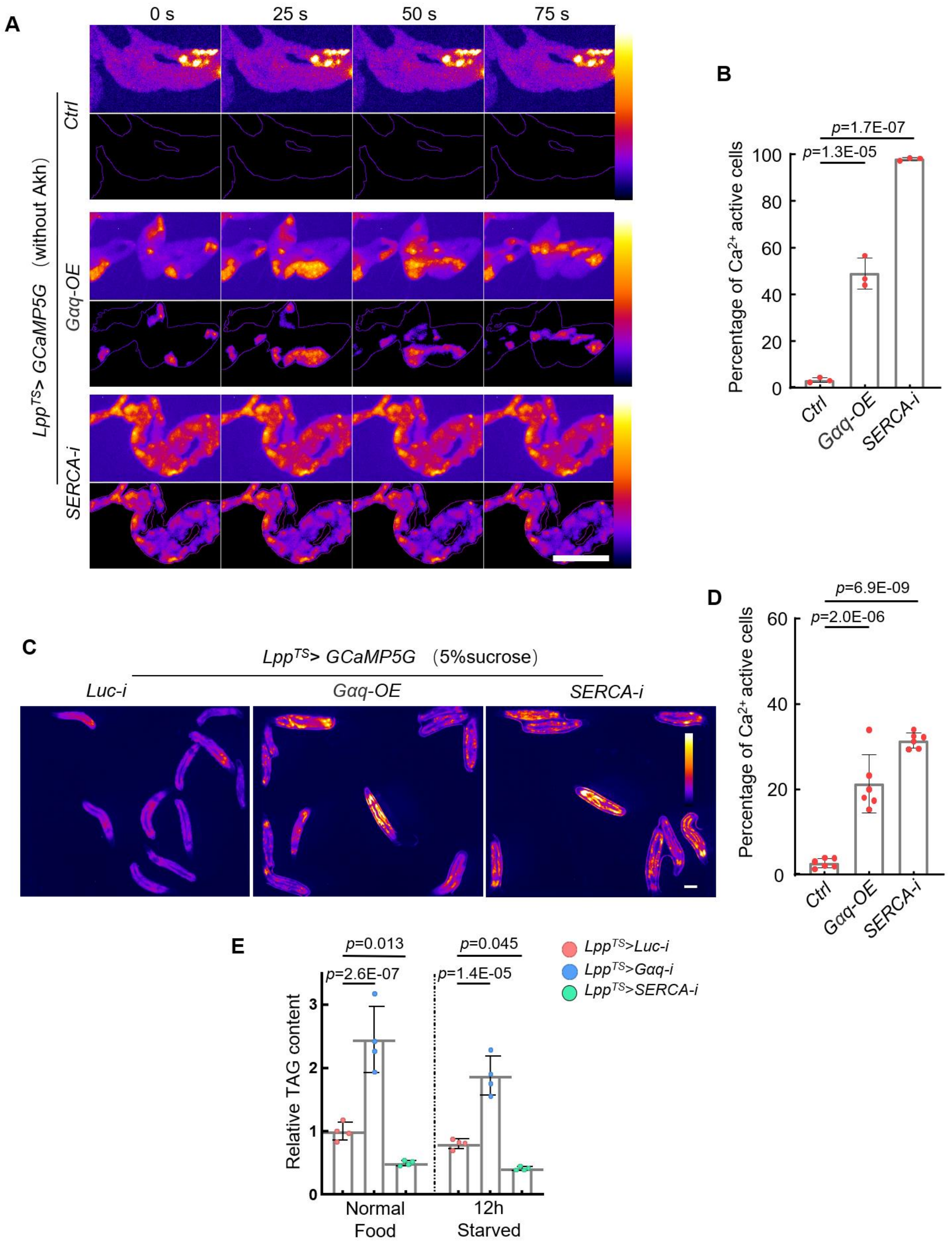

Supplementary Figure 4. Ca^{2+} activities are regulated by Gαq and SERCA in larval fat bodies. (A-B) Fat bodies were dissected from 3rd instar larvae expressing *GCaMP5G-T2A-mRuby* and indicated genes by *Lpp-Gal4*. One-way ANOVA with multiple comparisons was used for the significance test. The dynamic Ca^{2+} activities were highlighted in the lower panel by removing the constant background signal. N = 3, 3, 3 fat bodies

(**B**). **(C-D)** 3rd instar fly larvae expressing *GCaMP5G-T2A-mRuby* and indicated genes by *Lpp-Gal4* were kept on 2% agarose containing 5% sucrose and imaged under free-behaving conditions. Ca^{2+} activities in the fat bodies were quantified. *N* = 6, 6, 6 independent biological replicates (**D**). **(E)** Effects of Gαq or SERCA knockdown in fat bodies of adult flies on TAG metabolism. Temperature-sensitive *Gal80*^{TS} was used in the experiment to prevent early lethality. Larvae and adult flies were kept at 32°C for at least 3 days before the assay. *N* = 4, 4, 4, 4, 4, 4 independent biological replicates. Data were plotted as mean ± SD. Data were plotted as mean ± SD. Statistical significance was determined using Ordinary one-way ANOVA with Dunnett's multiple comparisons test in (**B**, **D**), and Ordinary two-way ANOVA with uncorrected Fisher's LSD was used in (**E**). Scale bars, 500 μm (**A**), 1000 μm (**C**). Source data are provided as a Source Data file.

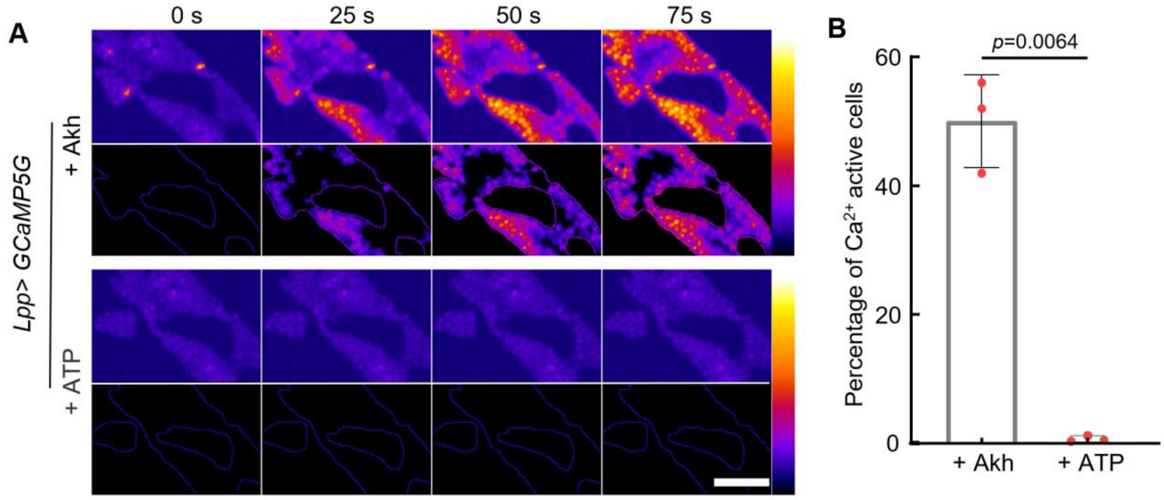

Supplementary Figure 5. Ca^{2+} waves are not triggered by ATP in the fly larval fat body. (A) Fat body expressing *GCaMP5G-T2A-mRuby* was cultured *ex vivo* and treated with 100 ng mL^{-1} synthesized AKH peptide or 5 μM ATP. Fat body failed to respond to ATP stimulation. The dynamic Ca^{2+} activities were highlighted in the lower panel by removing the constant background signal. **(B)** Ca^{2+} activities in the fat bodies were quantified. $N = 3$, 3 fat bodies. Statistical significance was determined using the paired two-tailed Student's t-test (**B**). Data were plotted as mean ± SD. Scale bars, 500 μm (**A**). Source data are provided as a Source Data file.

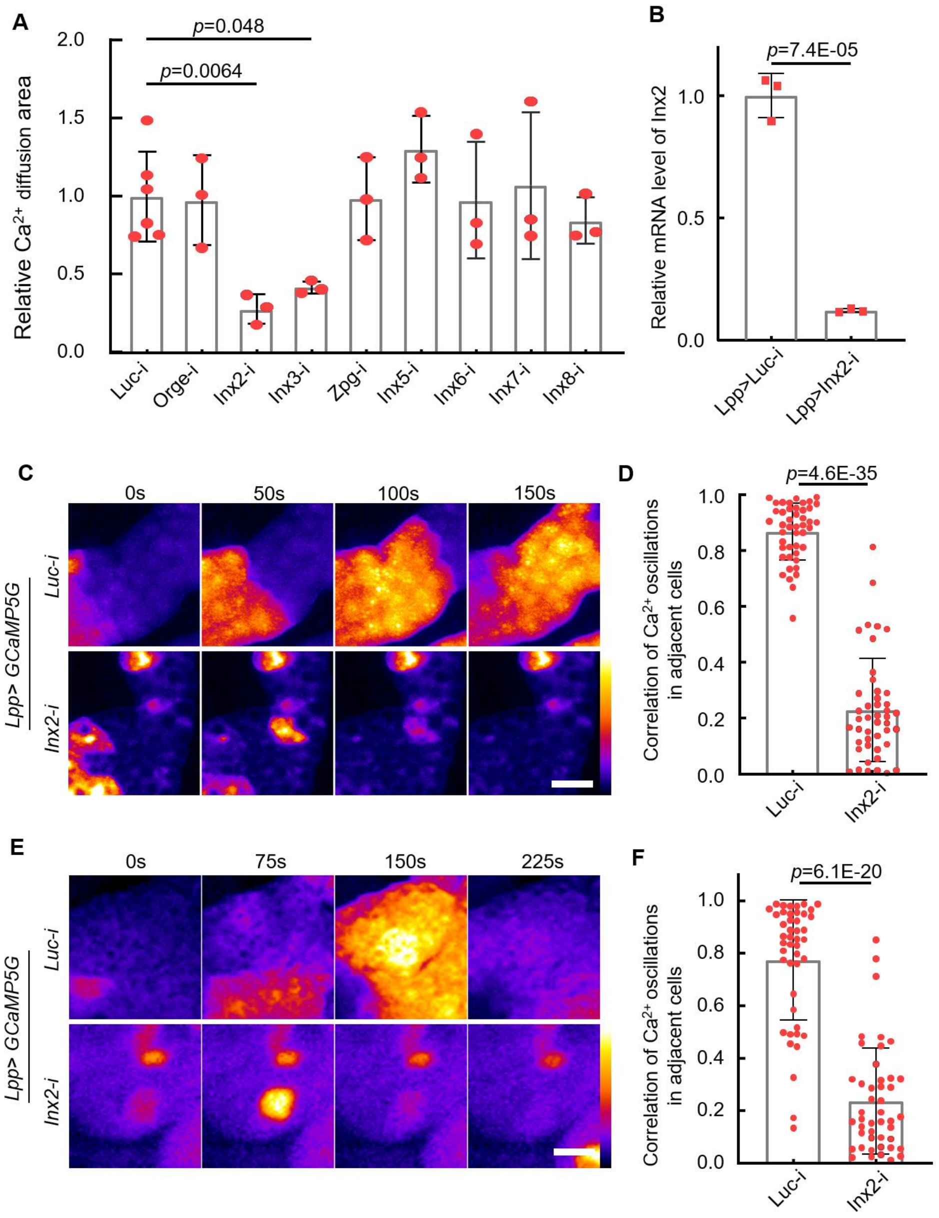

Supplementary Figure 6. Relative Ca^{2+} diffusion area after knocking down different gap junction proteins in the fly fat body. A. 3rd instar larvae expressing *GCaMP5G-T2A-mRuby* and indicated RNAi by *Lpp-Gal4* were kept at RT before dissection. Dissected fat bodies were treated with 100 ng mL^{-1} synthesized AKH peptide and the Ca^{2+} diffusion areas were quantified as described in the section. Only *Inx2* and *Inx3* knockdown significantly reduced the intercellular Ca^{2+} diffusion. N = 6, 3, 3, 3, 3, 3, 3, 3, 3 fat bodies. **B.** The qPCR data of *Inx2* mRNA level in the larval fat body with indicated genotype. N = 3, 3 independent biological replicates. **C-D.** Representative time-lapse images and quantification of the correlation between Ca^{2+} activities in adjacent fat cells. Larval fat cells with *Inx2* knockdown independently responded to AKH. Data was pooled from 3 independent experiments. N = 45, 45 pairs of adjacent cells (**D**). **E-F.** Representative time-lapse images and quantification of the correlation between Ca^{2+} activities in adjacent adult fat cells. Data was pooled from 3

independent experiments. *N* = 45, 45 pairs of adjacent cells (**F**). Ordinary one-way ANOVA with Dunnett's multiple comparisons test was used in (**A**), unpaired two-tailed Student's t-test was used in (**B**, **D**, **F**). Data were plotted as mean ± s.d.. Scale bars, 50 μm (**C**), 200 μm (**E**). Source data are provided as a Source Data file.

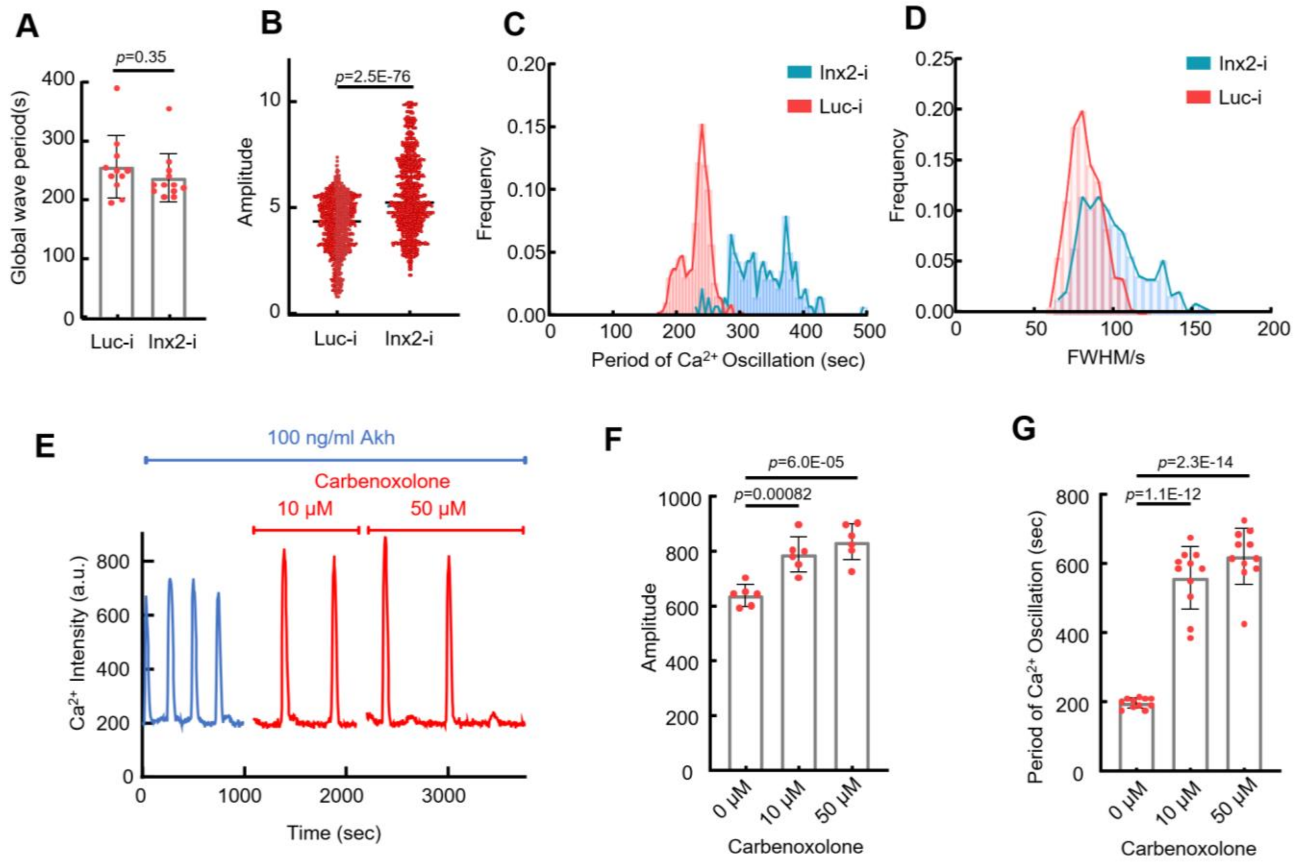

Supplementary Figure 7. Ca^{2+} activity in the larval fat body is affected by gap junction disruption. (A) The period of global Ca^{2+} waves was not affected by *Inx2* RNAi. Data was pooled from 3 independent experiments. *N* = 11, 12 oscillations. **(B-D)** Parameters of Ca^{2+} oscillation in *ex vivo* cultured larval fat body activated by 100 ng/mL AKH were quantified. The amplitude, period and peak width (full width at half maxima/FWHM) of the Ca^{2+} signal in the cells increased. Data was pooled from 3 independent experiments. *N* = 946, 940 oscillations **(B)**. **(E-G)** Gap junction inhibitor carbenoxolone triggered similar changes in the amplitude and period of Ca^{2+} waves, resembling those observed upon *Inx2* knockdown. A representative trace of Ca^{2+} dynamics from a single fat cell was plotted in **(E).** Data was pooled from 3 independent experiments. *N* = 6, 6, 6 fat cells **(F)**. *N* = 11, 11, 11 fat cells **(G)**. Data were plotted as mean ± SD. Paired two-tailed Student's t-test was utilized in **(A, B).** Ordinary one-way ANOVA with Dunnett's multiple comparisons test was used in **(F, G)**. Source data are provided as a Source Data file.

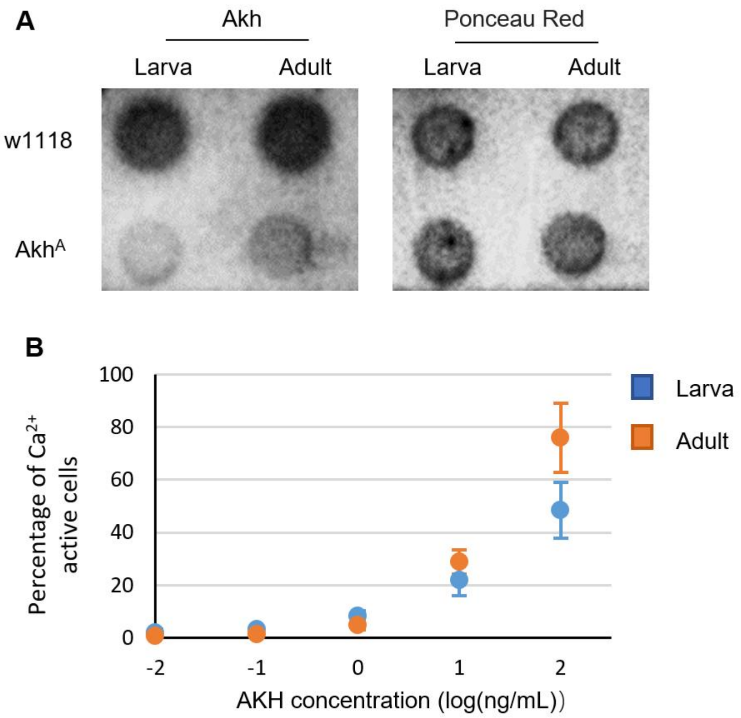

Supplementary Figure 8. Comparison of hemolymph AKH and AKH sensitivity of fat body between larva and adult. (A) Dot-blot assays indicate circulating Akh levels in the hemolymph of larvae and adults after starvation (48 hr). The data showed no significant difference in Akh concentration between the hemolymph of larval and adult flies. The Akh null mutant fly Akh^{A} was used as a negative control. **(B)** Dissected larval and adult fat bodies were treated with a culture medium containing different concentrations of AKH peptide. $N = 8$ fat bodies (Larva), 7 fat bodies (Adult). Data were plotted as mean ± SD and uncropped blots for **(A)** are provided in the Source Data file.

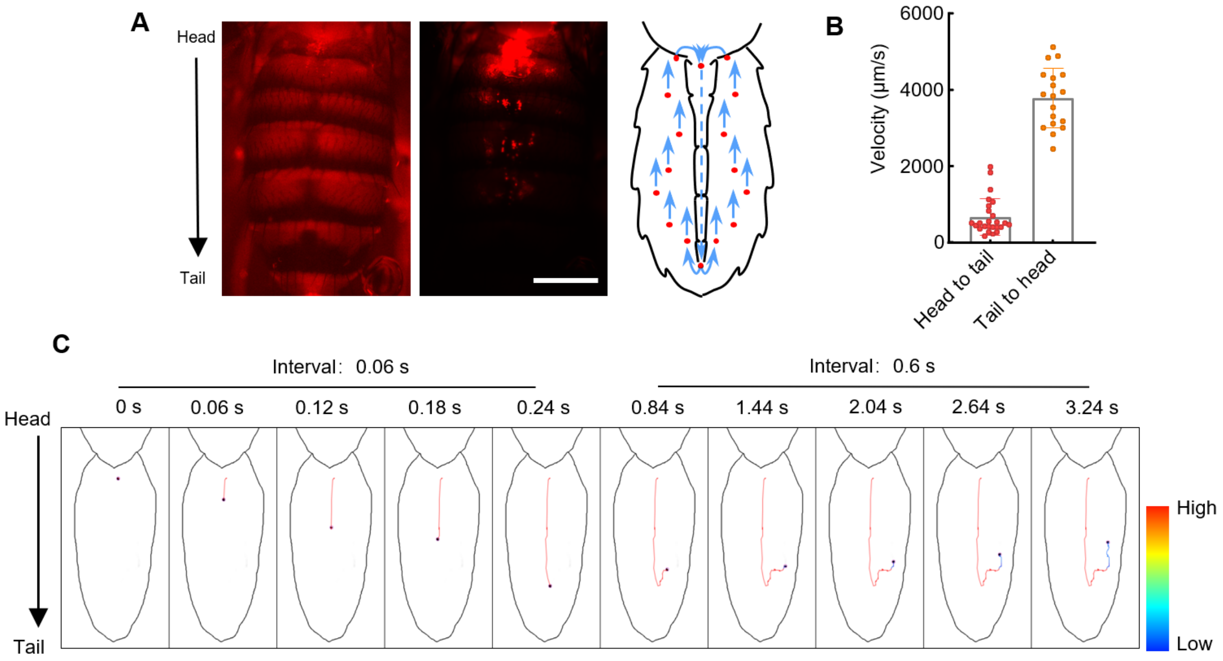

Supplementary Figure 9. Measurement of hemolymph flow speed in adult flies. (A-C) In the adult, fluorescent microspheres are rapidly pumped from the heart to the tail and then slowly flow to the anterior. Data were plotted as mean ± s.d.. Velocity from 5000 to 0 μm s^{-1} was color-coded linearly. Scale bars, 500 μm (**A**).

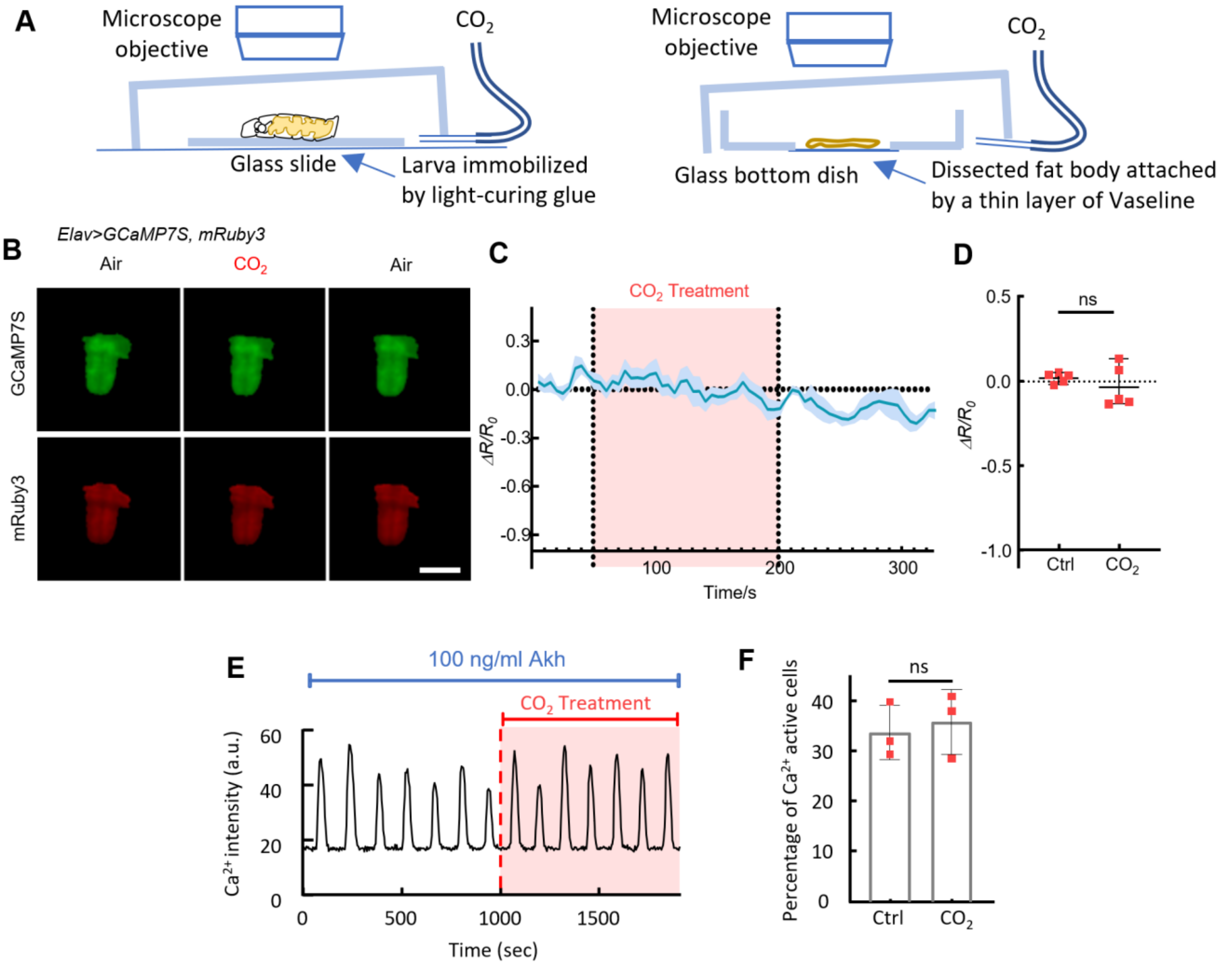

Supplementary Figure 10. CO_2 treatment does not affect the general brain Ca^{2+} activities or AKH-triggered Ca^{2+} activity in the fat body. (**A**) Illustration of setup for CO_2 treatment. (**B-D)** Whole brain activity before and after CO_2 application was monitored using *Elav>GCaMP7s, mRuby3*. The non-brain background fluorescent was removed using a brain-specific mask. Ca^{2+} activities before and after CO_2 application were quantified. Time courses of average Ca^{2+} signal from multiple larval brains (n=5) were plotted as mean ± s.e.m. (shaded region). Data was pooled from 5 independent experiments. N =5, 5 larvae **(D)**. **(E,F)** Ca^{2+} activity of the dissected fat body triggered by Akh was not affected by CO_2 treatment. Representative Ca^{2+} activity from a single fat cell was plotted in **(E)**. Data was pooled from 3 independent experiments. N =3, 3 fat bodies (F). Data in were plotted as mean ± S.E.M **(C),** and mean ± SD **(D).** Scale bars, 250 μm **(B)**. Paired two-tailed Student's t-test was utilized in **(D, F)**. Source data are provided as a Source Data file.

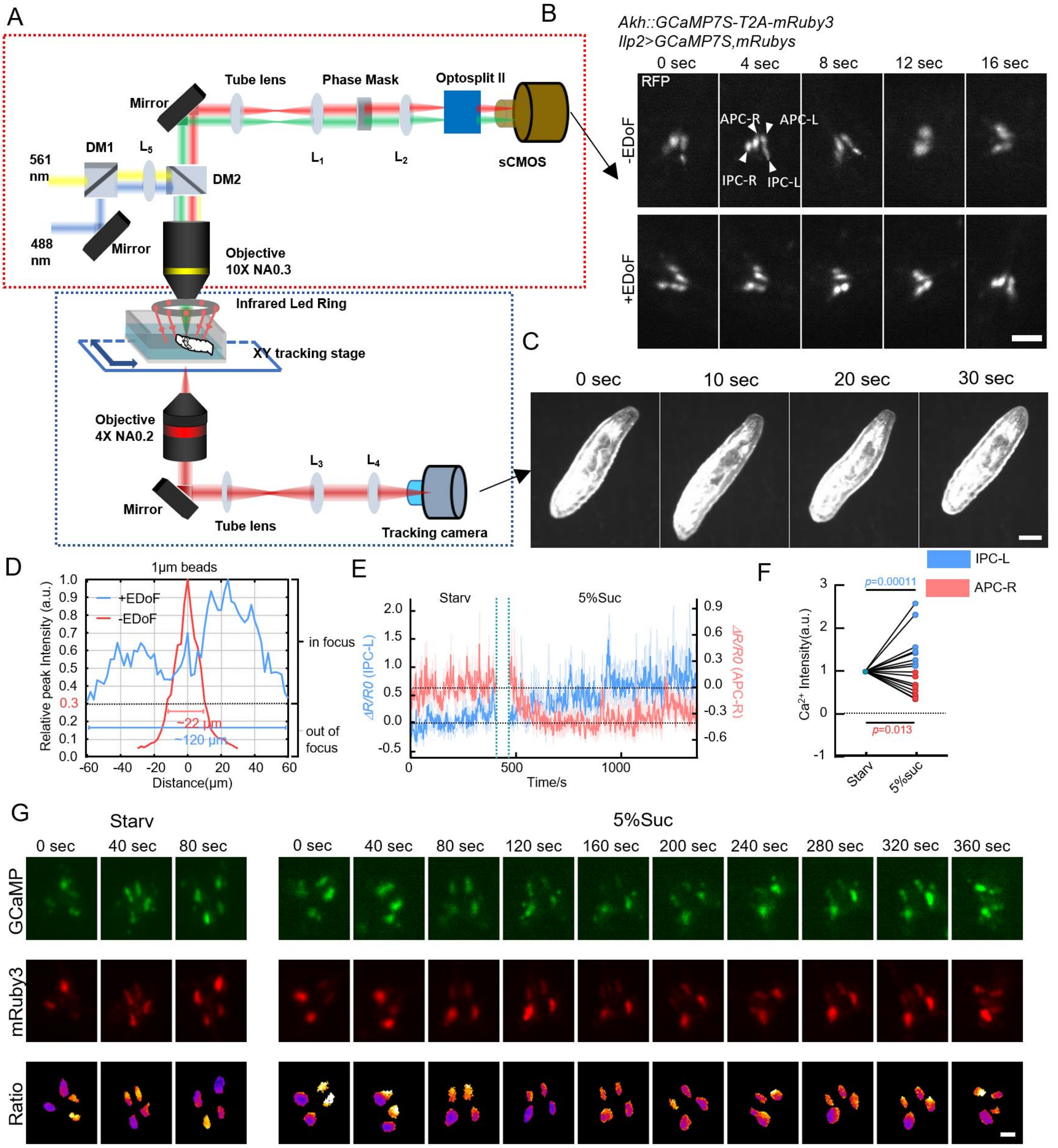

Supplementary Figure 11. Setups for imaging the Ca^{2+} activities in the APCs of free-behaving fly larvae. **(A)** The Extended-Depth-of-Field (EDoF) microscope has two modules: **1)** A darkfield imaging module (blue dotted box) equipped with a 4 x NA 0.2 air objective and a high-speed NIR camera to track and record free-behaving 1st instar larvae **(C)**; and **2)** A fluorescence imaging module (red dotted box) equipped with a 10X NA 0.3 air objective and a sCMOS camera, whose imaging surface is bisected by Optosplit II that allows simultaneous recording of two fluorescence signals (calcium-sensitive GCaMP and calcium-insensitive RFP as reference). The microscope's aspheric phase mask is placed on the conjugate plane of the pupil plane of the

objective, i.e., the back focal plane of the achromatic lens. In this way, the PSF (point spread function) of the system is modified to extend the effective depth of field (we set the out-of-focus standard as a relative peak intensity of 0.3 for 1 μm fluorescent beads) by about 5 times **(D)**. **(B)** Representative RFP images of the APCs and IPCs of free-behaving larvae between –EDoF and +EDoF. After using EDoF technology, out-of-focus situations were reduced. **(E)** Representative Ca^{2+} activities in the APCs (red) and IPCs (blue) of free-behaving larvae transferred between starvation and 5%sucrose. Time courses of average Ca^{2+} signal from multiple larvae (n=8) were plotted as mean ± s.e.m. (shaded region) (N=9). **(F)** Quantification of Ca^{2+} activities in the APCs of free-behaving larvae. Data was pooled from 9 independent experiments. *N* =9, 9 larvae. **(G)** Representative Ca^{2+} images of the APCs of free-behaving larvae. Scale bars, 40 μm **(B)**, 200 μm **(C)**, 20 μm **(G)**. Statistical significance was determined using paired two-tailed Student's t-test **(F)**. Source data are provided as a Source Data file.

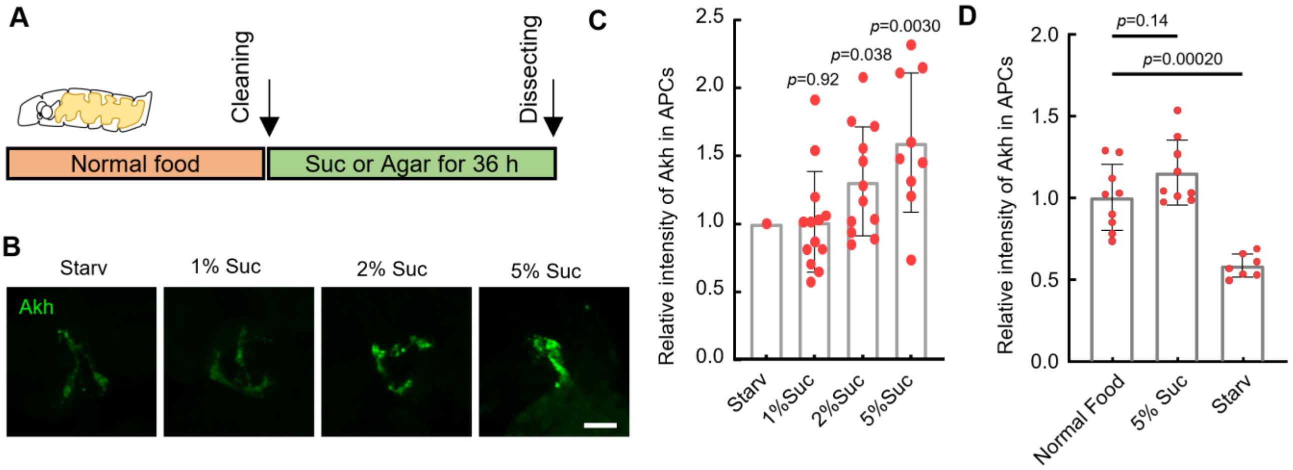

Supplementary Figure 12. AKH is released after starvation. (A) Early 3rd instar larvae were kept on 2% agarose (Starvation) or 2% agarose + different centration of sucrose (Suc) for 36 hours. (**B**) The remaining AKH in the APCs of the larvae were stained with anti-AKH antibody. **(C)** Quantification of AKH signals in the APCs. *N* = 9 brains (1%Suc), 12 brains (2%Suc), 13 brains (5%Suc). **(D)** Quantifications of the fluorescent intensity in APCs of fly larvae fed on Normal food, 5% sucrose, or no nutrient (1% agarose alone). *N* = 9 brains (1%Suc), 9 brains (2%Suc), 7 brains (5%Suc). Data were plotted as mean ± SD. Scale bars, 25 µm (**B**). Unpaired two-tailed Student's t-test is used in (**C**), Ordinary one-way ANOVA with Dunnett's multiple comparisons test was used in (**D**). Source data are provided as a Source Data file.

Supplementary Table 1: Fly stocks used in this study

Genotypes	Source	Identifier
w1118	Lab Stock	
Canton S	Lab Stock	
$AkhR^{null}$	Bloomington Stock Center	BDSC_80937
Akh^{A}	From Dr. Song Wei	
tub-$GAL80^{TS}$; Lpp-gal4	Lab Stock	
Akh-gal4	Bloomington Stock Center	BDSC_25684
Akh-gal4 (attP8)	This study	N/A
Akh::GCaMP7S-T2A-mRuby3 (attP2)	This study	N/A
Ilp2-gal4	Bloomington Stock Center	BDSC_37516
Fb-Gal4	From Dr. Jun-yuan Ji	
UAS-GCaMP5G-T2A-mRuby3 (attP2)	This study	N/A
UAS-Kir2.1	Bloomington Stock Center	BDSC_6596
UAS-Akh-RNAi	Bloomington Stock Center	BDSC_34960
UAS-GCaMP5G	Bloomington Stock Center	BDSC_42037
UAS-Akh	Bloomington Stock Center	BDSC_27343
UAS-Gαq-RNAi	Bloomington Stock Center	BDSC_33765
UAS-Luc-RNAi	Bloomington Stock Center	BDSC_35788
UAS-Gαq	Bloomington Stock Center	BDSC_30784
UAS-Serca-RNAi	Bloomington Stock Center	BDSC_25928
UAS-AkhR-RNAi	Bloomington Stock Center	BDSC_29577
UAS-Ogre-RNAi	Bloomington Stock Center	BDSC_27283
UAS-Inx2-RNAi	Bloomington Stock Center	BDSC_29306
UAS-Inx3-RNAi	Bloomington Stock Center	BDSC_30501
UAS-Zpg-RNAi	Bloomington Stock Center	BDSC_27674
UAS-Inx5-RNAi	Bloomington Stock Center	BDSC_28042
UAS-Inx6-RNAi	Bloomington Stock Center	BDSC_31889
UAS-Inx7-RNAi	Bloomington Stock Center	BDSC_26297
UAS-ShakB-RNAi	Bloomington Stock Center	BDSC_27292

Supplementary Table 2：Sequences of neuropeptides used in this study

**Name**	**Sequence**
AKH	QLTFSPDW-amide
APK	SVAALAAQGLLNAPK
Ast-A	SRPYSFGL-amide
Ast-C	QVRYRQCYFNPISCF-amide
CAPA-PVK	GANMGLYAFPRV-amide
CAPA-PK	TGPSASSGLWFGPRL-amide
CCAP	PFCNAFTGC-amide
CCHamide-1	SCLEYGHSCWGAH-amide
CCHamide-2	GCQAYGHVCYGGH-amide
Corazonin	QTFQYSRGWTN-amide
DH31	TVDFGLARGYSGTQEAKHRMGLAAANFAGGP-amide
DH44	NKPSLSIVNPLDVLRQRLLLEIARRQMKENSRQVELNRAILKNV-amide
Drosulfakinins	FDDYGHMRF-amide
DSK-2	GGDDQFDDYGHMRF-amide
ETH	DDSSPGFFLKITKNVPRL-amide
FMRFa	SPKQDFMRF-amide
Hug	SVPFKPRL-amide
IPNamide	NVGTLARDFQLPIPN-amide
Leucokinin	NSVVLGKKQRFHSWG-amide
Mip	AWKSMNVAW-amide
MTYamide	YIGSLARAGGLMTY-amide
Myosuppressin	TDVDHVFLRF-amide
NEF	TKAQGDFNEF
NPF	SNSRPPRKNDVNTMADAYKFLQDLDTYYGDRARVRF
Pdf	NSELINSLLSLPKNMNDA-amide
SHA	VVSVVPGAISHA
SIFa	AYRKPPFNGSIF-amide
sNPF	SPSLRLRF-amide
Tachykinin-1	APTSSFIGMR-amide
VQQ	NLGALKSSPVHGVQQ
VVIamide	SVHGLGPVVI-amide
YSY	pQYYYGASPYAYSGGYYDSPYSY

Supplementary Table 3 : Parameters used in the computational model and numerical simulation

Parameter	Description	Value
k_f	Maximal rate of Ca^{2+} release through IPR.	0.888 s^{-1}
K_1	Rate constant characterizing IP3 binding to IPR.	0.13 μM
K_2	Rate constant characterizing Ca^{2+} binding to activating site of IPR.	82 μM
V_s	Maximum rate of SERCA pump.	720 $\mu M s^{-1}$
K_s	Half activation constant of Ca^{2+} binding to SERCA pump.	100 μM
y	Fraction of inactivated IPR.	0.3
k_{leak}	Rate constant of Ca^{2+} leak from ER.	0.016 s^{-1}
α_1	IP3 contributing to the flux into the cell.	56 s^{-1}
α_0	Constant flux into the cell.	8 $\mu M s^{-1}$
D_c	Diffusion constant of the Ca^{2+} through gap junctions.	16 $\mu m^2 s^{-1}$
γ	Ratio of effective volume cytoplasm/ER.	2
k_A	Ratio of steady concentration IP3/AKH.	0.0005
p_0	Basic IP3 concentration.	0.12 μM
μ	Delay rate of AKH.	0.016 s^{-1}
v_{tr}	Transport speed of AKH.	36 $\mu m s^{-1}$
D_A	Diffusion constant of AKH.	120 $\mu m^2 s^{-1}$
σ_c	Fluctuation constant of Ca^{2+} in cytoplasm.	20 μM
σ_a	Fluctuation constant of AKH.	1.6 nM
$L_{x,larva}$	Width of larvae.	1000 μm
$L_{y,larva}$	Length of larvae.	3500 μm
$L_{x,adult}$	Width of adults.	1000 μm
$L_{y,adult}$	Length of adults.	1600 μm
dl_{larva}	Length of a larvae cell (assume cells as squares).	40 μm
dl_{adult}	Length of an adult cell (assume cells as squares).	20 μm
$C_{A0,larva}$	Basic AKH concentration in larvae.	0
$C_{A0,adult}$	Basic AKH concentration in adult.	10 nM
$C_{A,pulse}$	Pulse AKH concentration in larvae.	100 nM
T_{pulse}	Period of AKH pulse.	300 s
