# Supplementary material for: Dietary amino acids promote glucagon-like hormone release to generate global calcium waves in adipose tissues in Drosophila
Source: Nat Commun. 2025 Jan 2;16:247. doi: 10.1038/s41467-024-55371-y (PMC11696257; doi:10.1038/s41467-024-55371-y)
Supplement: Supplementary file 2 — Description of Additional Supplementary Files [file 41467_2024_55371_MOESM2_ESM.pdf]

Description of Additional Supplementary Files

File name: Supplementary Data 1
Description: List of complete fly genotypes used in each figure.

File name: Supplementary Movie 1
Description: Ca^{2+} activities in immobilized 3rd instar larva with *Lpp>GCaMP5G*.

File name: Supplementary Movie 2
Description: Ca^{2+} activities in the isolated larval fat body (*Fb>GCaMP5G*) cultured with or without the presence of brain tissues. The dynamic ICWs were highlighted by removing the constant background signal in the lower panel.

File name: Supplementary Movie 3
Description: Ca^{2+} activities in isolated fat body tissue from control, *AkhR* mutant, and *Gαq* knockdown larvae (*Lpp> GCaMP5G-T2A-mRuby3*). The dynamic ICWs were highlighted by removing the constant background signal in the lower panel.

File name: Supplementary Movie 4
Description: Ca^{2+} activities in the fat body of free-behaving control and *AkhR* mutant larvae with *Lpp> GCaMP5G-T2A-mRuby3*.

File name: Supplementary Movie 5
Description: Ca^{2+} activities in the isolated fat body from control and *Inx2* knockdown larvae with *Lpp> GCaMP5G-T2A-mRuby3*. The dynamic ICWs were highlighted by removing the constant background signal in the lower panel.

File name: Supplementary Movie 6
Description: Ca^{2+} activities in immobilized 3rd instar larvae (*Lpp>GCaMP5G-T2A-mRuby3*) with control, *Inx2-RNAi*, *AkhR* mutation and *Akh* overexpression.

File name: Supplementary Movie 7
Description: Ca^{2+} activities in the cultured fat body from control and *Inx2-RNAi* adult flies with *Lpp> GCaMP5G-T2A-mRuby3*.

File name: Supplementary Movie 8
Description: Ca^{2+} activities in immobilized control, *Inx2-RNAi*, and *AkhR* mutant adult flies with *Lpp>GCaMP5G*.

File name: Supplementary Movie 9
Description: Immobilized 3rd instar larvae with *Lpp> GCaMP5G-T2A-mRuby3* were treated with or without Chloroform.

File name: Supplementary Movie 10
Description: Tracking of fluorescent beads injected into the 3rd instar larvae and adult flies (Canton S).

File name: Supplementary Movie 11
Description: Simulation of global ICWs in 3rd instar larvae with and without gap junction knockdown.

File name: Supplementary Movie 12
Description: Simulation of local ICWs in the adult flies with and without gap junction knockdown.

File name: **Supplementary Movie 13**
Description: Ca^{2+} activities in the fat body of free-behaving larvae with *Lpp>GCaMP5G-T2A-mRuby3* as they were transferred from a 2% sucrose diet to indicated diets.

File name: Supplementary Movie 14
Description: Ca^{2+} activities in the APCs (*Akh>GCaMP5G-T2A-mRuby3*) of 1st instar larvae when transferred between indicated diets.

File name: Supplementary Movie 15
Description: Ca^{2+} activities in the fat body (*Lpp> GCaMP5G-T2A-mRuby3*) treated with brain-conditioned HL6 (AA-) medium supplemented with indicated amino acids.
