# Supplementary material for: Dietary amino acids promote glucagon-like hormone release to generate global calcium waves in adipose tissues in Drosophila
Source: Nat Commun. 2025 Jan 2;16:247. doi: 10.1038/s41467-024-55371-y (PMC11696257; doi:10.1038/s41467-024-55371-y)
Supplement: Supplementary file 19 — Reporting Summary [file 41467_2024_55371_MOESM19_ESM.pdf]

nature portfolio

Corresponding author(s): Li He, Norbert Perrimon, Yuansheng Cao

Last updated by author(s): Nov 27, 2024

Reporting Summary

Nature Portfolio wishes to improve the reproducibility of the work that we publish. This form provides structure for consistency and transparency in reporting. For further information on Nature Portfolio policies, see our Editorial Policies and the Editorial Policy Checklist.

Statistics

For all statistical analyses, confirm that the following items are present in the figure legend, table legend, main text, or Methods section.

n/a	Confirmed	
☐	☒	The exact sample size (*n*) for each experimental group/condition, given as a discrete number and unit of measurement
☐	☒	A statement on whether measurements were taken from distinct samples or whether the same sample was measured repeatedly
☐	☒	The statistical test(s) used AND whether they are one- or two-sided<br>*Only common tests should be described solely by name; describe more complex techniques in the Methods section.*
☒	☐	A description of all covariates tested
☒	☐	A description of any assumptions or corrections, such as tests of normality and adjustment for multiple comparisons
☐	☒	A full description of the statistical parameters including central tendency (e.g. means) or other basic estimates (e.g. regression coefficient) AND variation (e.g. standard deviation) or associated estimates of uncertainty (e.g. confidence intervals)
☐	☒	For null hypothesis testing, the test statistic (e.g. *F*, *t*, *r*) with confidence intervals, effect sizes, degrees of freedom and *P* value noted<br>*Give P values as exact values whenever suitable.*
☒	☐	For Bayesian analysis, information on the choice of priors and Markov chain Monte Carlo settings
☒	☐	For hierarchical and complex designs, identification of the appropriate level for tests and full reporting of outcomes
☐	☒	Estimates of effect sizes (e.g. Cohen's *d*, Pearson's *r*), indicating how they were calculated

Our web collection on statistics for biologists contains articles on many of the points above.

Software and code

Policy information about availability of computer code

Data collection	No unpublished software was used. Images were acquired on Leica-M205 FCA, Leica DMi 8 microscope, and home-made EDoF microscope. Leica Application Suite X software and Micro-manager software were used for data collection.
Data analysis	The images were analyzed utilizing FIJI (Imaged), MathWorks MATLAB, and GraphPad Prism. Computational simulations were carried out with the MathWorks MATLAB software. For details regarding the algorithms and parameters employed in the modeling process, please refer to Supplementary methods and Supplementary Table 3.

For manuscripts utilizing custom algorithms or software that are central to the research but not yet described in published literature, software must be made available to editors and reviewers. We strongly encourage code deposition in a community repository (e.g. GitHub). See the Nature Portfolio guidelines for submitting code & software for further information.

Data

Policy information about availability of data

All manuscripts must include a data availability statement. This statement should provide the following information, where applicable:

- Accession codes, unique identifiers, or web links for publicly available datasets
- A description of any restrictions on data availability
- For clinical datasets or third party data, please ensure that the statement adheres to our policy

All codes used for modeling of this study are deposited in the Github and Zenository. ttps://github.com/Sy-Luo/HeLab_CalciumWave_Simulation_2024 and the open-access repository Zenodo with DOI: 10.5281/zenodo.14175603.

Research involving human participants, their data, or biological material

Policy information about studies with human participants or human data. See also policy information about sex, gender (identity/presentation), and sexual orientation and race, ethnicity and racism.

Reporting on sex and gender	N/A
Reporting on race, ethnicity, or other socially relevant groupings	N/A
Population characteristics	N/A
Recruitment	N/A
Ethics oversight	N/A

Note that full information on the approval of the study protocol must also be provided in the manuscript.

Field-specific reporting

Please select the one below that is the best fit for your research. If you are not sure, read the appropriate sections before making your selection.

☒ Life sciences ☐ Behavioural & social sciences ☐ Ecological, evolutionary & environmental sciences

For a reference copy of the document with all sections, see nature.com/documents/nr-reporting-summary-flat.pdf

Life sciences study design

All studies must disclose on these points even when the disclosure is negative.

Sample size	Sample size was chosen based on published literature or determined by a power calculation.
Data exclusions	No data were excluded from analyses.
Replication	All experiments were repeated with at least 3 independent biological replicates as indicated in the figure legends.
Randomization	Samples were allocated into experimental groups based on their genotypes. Randomization was not relevant to this study.
Blinding	The investigators were not blinded during data collection. All fly crosses were marked with different phenotypic markers for genotyping. Investigators were not blinded to the group allocation, in accordance with standard practices in the field.

Reporting for specific materials, systems and methods

We require information from authors about some types of materials, experimental systems and methods used in many studies. Here, indicate whether each material, system or method listed is relevant to your study. If you are not sure if a list item applies to your research, read the appropriate section before selecting a response.

Materials & experimental systems

n/a	Involved in the study
☐	☒ Antibodies
☒	☐ Eukaryotic cell lines
☒	☐ Palaeontology and archaeology
☐	☒ Animals and other organisms
☒	☐ Clinical data
☒	☐ Dual use research of concern
☒	☐ Plants

Methods

n/a	Involved in the study
☒	☐ ChIP-seq
☒	☐ Flow cytometry
☒	☐ MRI-based neuroimaging

Antibodies

Antibodies used	Anti-AKH antibody for dot-blot was from Dr. Wei Song. Anti-AKH for immunostaining was generated by ABclonal.
Validation	The antibody has been validated by previously published literature and immunofluorescence staining of fly tissue in this study.

Animals and other research organisms

Policy information about studies involving animals; ARRIVE guidelines recommended for reporting animal research, and Sex and Gender in Research

Laboratory animals	2nd and 3rd instar larva and 5-10 day old adults of Drosophila melanogaster flies were used in this study. A full list of fly lines used in this study was listed in the Supplementary Table 1.
Wild animals	No wild animals were used in this study.
Reporting on sex	Experiments were conducted on male and female larval flies as well as female adult flies.
Field-collected samples	No field-collected samples were used in this study.
Ethics oversight	Ethical approval was not required for Drosophila Studies.

Note that full information on the approval of the study protocol must also be provided in the manuscript.

Plants

Seed stocks	n/a
Novel plant genotypes	n/a
Authentication	n/a
